# Structural and Functional Characterization of CreFH1, the Frataxin Homolog from *Chlamydomonas reinhardtii*

**DOI:** 10.3390/plants11151931

**Published:** 2022-07-26

**Authors:** Agustina Terenzi, Maria A. Pagani, Diego F. Gomez-Casati, Maria V. Busi

**Affiliations:** Centro de Estudios Fotosintéticos y Bioquímicos (CEFOBI-CONICET), Universidad Nacional de Rosario, Rosario 2000, Argentina; terenzi@cefobi-conicet.gov.ar (A.T.); pagani@cefobi-conicet.gov.ar (M.A.P.)

**Keywords:** frataxin, *Chlamydomonas*, algae

## Abstract

Frataxin plays a key role in cellular iron homeostasis of different organisms. It has been implicated in iron storage, detoxification, delivery for Fe-S cluster assembly and heme biosynthesis. However, its specific role in iron metabolism remains unclear, especially in photosynthetic organisms. To gain insight into the role and properties of frataxin in algae, we identified the gene CreFH1, which codes for the frataxin homolog from *Chlamydomonas reinhardtii*. We performed the cloning, expression and biochemical characterization of CreFH1. This protein has a predicted mitochondrial transit peptide and a significant structural similarity to other members of the frataxin family. In addition, CreFH1 was able to form a dimer in vitro, and this effect was increased by the addition of Cu^2+^ and also attenuated the Fenton reaction in the presence of a mixture of Fe^2+^ and H_2_O_2_. Bacterial cells with overexpression of CreFH1 showed increased growth in the presence of different metals, such as Fe, Cu, Zn and Ni and H_2_O_2_. Thus, results indicated that CreFH1 is a functional protein that shows some distinctive features compared to its more well-known counterparts, and would play an important role in response to oxidative stress in *C. reinhardtii.*

## 1. Introduction

Frataxin is a highly conserved protein present in most organisms, including bacteria, fungi, mammal, plants and algae. Its deficiency was initially described in humans as the primary cause of Friedreich’s ataxia, a cardio- and neurodegenerative disease characterized by oxidative stress and iron accumulation in mitochondria [1,2,3,4]. It was reported that frataxin plays an essential role in mitochondria biogenesis and is required for cellular iron homeostasis regulation in different organisms, iron-sulfur cluster assembly, heme metabolism, oxidative phosphorylation, oxidative stress and NO signaling [5,6,7,8,9,10,11,12,13,14,15,16,17]. Recent studies have proposed frataxin as a regulator of ferroptosis by modulating iron homeostasis and mitochondrial function [18]. Nevertheless, it is possible that frataxin may also have specific functions within each organism or cellular tissue. Even though it has been implicated in diverse important processes, its exact molecular function remains unclear at present.

Previous studies in yeast showed that frataxin mediates the delivery of iron Fe^2+^ to the protein Isu1 to assemble Fe-S clusters [19]. In humans, yeast and *Arabidopsis thaliana* frataxin is able to interact with other proteins from the Fe-S cluster biosynthesis pathway forming multiproteic complexes [20,21,22,23,24,25,26]. Frataxin also provides the iron for hemo cluster biosynthesis, interacting with ferrochelatase in humans and yeast [27,28] or as a part of the plant ISC complex [29]. Furthermore, several studies associate frataxin with cellular redox control, iron detoxification and protection against oxidative stress [15,30,31].

Although many studies have been conducted on human and yeast frataxin, there are few reports on the characterization of frataxins from photosynthetic organisms. We previously described the existence of two frataxin isoforms in maize, with localization in chloroplasts and mitochondria and the presence of only one frataxin isoform (AtFH), but dually localized in both organelles in *Arabidopsis thaliana* [32,33,34,35]. AtFH can complement *Saccharomyces cerevisiae* null mutant cells for the frataxin gene YFH (*Δyfh*), restoring its respiratory rate and decreasing its sensibility to oxidant reagents [32,36]. This suggests that AtFH could be involved in mitochondrial respiration and in the oxidative stress response. Previous studies showed that *atfh* homozygote null mutants in *Arabidopsis* are lethal at embryogenesis level, suggesting that frataxin could have an essential role in plants [37]. AtFH deficient lines presented a high content of mitochondrial iron, increased ROS production, induction of oxidative stress marker genes and decreased activity of mitochondrial and chloroplastic Fe-S enzymes [33,34]. In addition, the deficiency in AtFH caused an alteration in the transcript levels of proteins involved in heme biosynthesis, lower heme content, and reduced activity of hemoproteins, such as catalase. These results suggest that AtFH is involved in heme biosynthesis and hemoprotein biogenesis in plants [13]. However, its specific role in iron metabolism remains unclear, especially in photosynthetic organisms.

In recent years, the unicellular green algae *Chlamydomonas reinhardtii* has been extensively used to study iron metabolism. This microalga has a compact and sequenced genome, rapid growth in defined medium and availability of a wide variety of well-established molecular biology tools [38,39]. Previous bioinformatic analysis of *C. reinhardtii* genome showed a remarkable conservation of the proteins involved in the Fe-S biosynthesis pathway; this alga contains the same set of components of the mitochondria ISC, export, CIA and chloroplast SUF machinery as *Arabidopsis thaliana*, with minor exceptions [40,41]. Many ISC machinery genes in plants are present in multiple copies; however, some of these genes, such as frataxin, exist as a single copy in *Chlamydomonas* [40].

As mentioned, there are few reports on the characterization of plant frataxin homologs. However, no information is available about any algae counterpart. Thus, in order to expand the knowledge on the structure and function of the components of the ISC machinery in algae, we identified the presence of the *CreFH1* gene, which codes for a putative frataxin homolog from *C. reinhardtii*. *CreFH1* was cloned and heterologously expressed, and the protein was purified and characterized. Protein modeling showed that CreFH1 shares structural similarity with other frataxin homologs. In addition, the presence of Cu^2+^ and other metals, such as Fe^3+^ and Zn^2+^, increased the oligomerization of CreFH1. Moreover, *E. coli* cells expressing CreFH1 were able to grow better in the presence of these metals and other oxidative stress agents. Our results indicate that CreFH1 is a functional protein of *C. reinhardtii,* and might be involved in metal homeostasis, protection against metal oxidative damage and/or the maintenance of cellular redox state.

## 2. Results

### 2.1. Gene Structure, Protein Sequence Analysis and Homology Modelling

**Figure 1:**
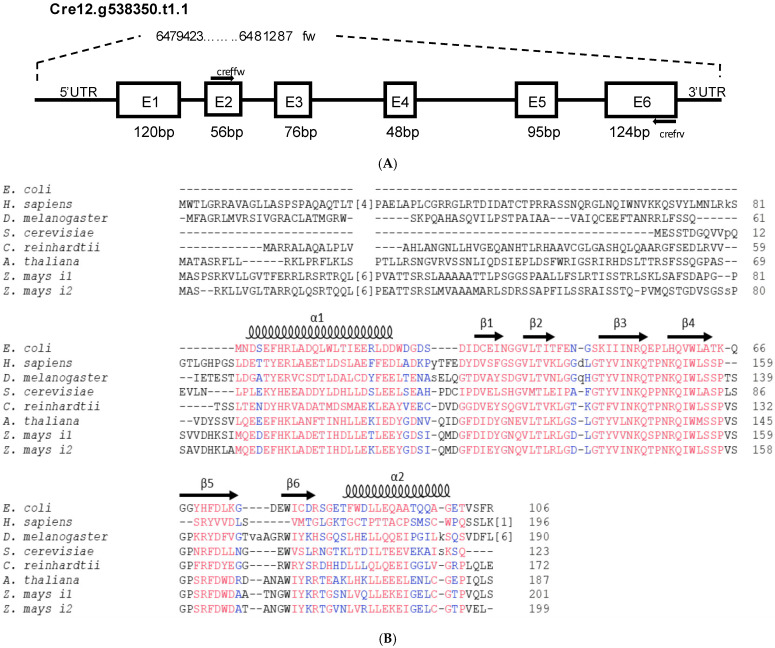
Gene structure organization of *Chlamydomonas reinhardtii* frataxin. (**A**) Structure of the gene coding the *C. reinhardtii* frataxin homologue (Cre12.g538350.t1.1, abbrev. *CreFH1*). *CreFH1* is composed of 6 exons and 5 introns. The arrows correspond to the oligonucleotides used in the molecular analyses. 5’UTR and 3’UTR correspond to the 5’ and 3’ untranslated regions. (**B**) Alignment of the amino acid sequences of frataxin homologues from different organisms. The protein sequence of CreFH1 (accession number XP_042918788.1) was compared with CyaY from *E. coli* (accession number NP_418251) and frataxin homologs from *H. sapiens* (accession number NP_000135.2), *D. melanogaster* (NP_511094), *S. cerevisiae* (accession number NP_010163.1), *A. thaliana* (accession number NP_192233.2) and both *Z. mays* isoforms (ZmFH1, NP_001150732 and ZmFH2, NP_001146272). Positions with a high degree of conservation are indicated in red, and positions with a lower degree of conservation are indicated in blue. Secondary structure is indicated on the *E. coli* sequence with coils (α-helices) and arrows (β-sheets). The arrow indicates the position of the transit peptide cleavage predicted with TargetP-2.0 Server [45].

Based on the information obtained from Phytozome v12 [42], we found the *CreFH1* gene (Cre12.g538350t1.1) in chromosome 12 of *C. reinhardtii.* This gene contains 1864 bp from the 5′ to the 3′ UTR regions, and it is composed of six exons and five introns, which produce a transcript with a 519 bp CDS (Figure 1A). The gene codes for a 172 amino acid putative frataxin homolog protein with an estimated molecular mass of 19 kDa. Domain identification was performed using CD-Search [43,44]. We found that CreFH1 contains a frataxin-like domain between the amino acid region 63-169. In addition, the analysis using TargetP-2.0 Server [45] suggested the presence of a mitochondrial transit peptide of 52 amino acids (score 0.88). Thus, the mature form of CreFH1 would contain 120 amino acids with a molecular mass of 13.56 kDa, and it would be targeted to that organelle.

The amino acid sequence of CreFH1 shares 25% and 41% identity with the homolog proteins from *Escherichia coli* (CyaY, EDU66360.1) and *Saccharomyces cerevisiae* (YFH, ONH78464.1), respectively, and about 47% to 51% identity with other characterized frataxins, such as those from humans (HsFH, AAH48097.1), *Drosophila melanogaster* (DmFH, NP_511094.1), *Arabidopsis thaliana* (AtFH, NP_192233.2) and both proteins from *Zea mays* (ZmFH-1 and -2, NP_001146272.1 and NP_001150732.1) (Figure 1B). In addition, we found that CreFH1 contains 12 acidic residues, most of them located in conserved positions, in the α1 and β1 regions of the protein. These data indicate that CreFH1 is highly conserved among different species.

To understand the possible roles of CreFH1, it is relevant to analyze its 3D structure. Solution and crystal structures have been reported for different frataxin homologs, such as HsFH, bacterial CyaY and YFH [46,47,48,49,50]. Several structural studies showed that the characteristic frataxin folding consists of a compact α-β sandwich composed of two N- and C-terminal α-helix and seven β-strands in the central region of the protein. Analysis of CreFH1 using Jpred 4 [51] predicted a similar fold with two α-helix, but only six β-strands in the central region of the protein (Figure 1B).

**Figure 2:**
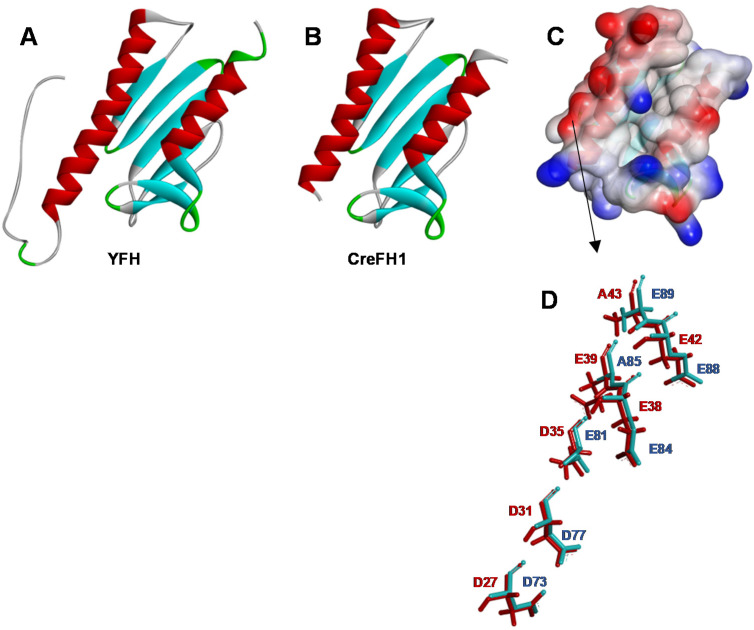
Homology modeling of CreFH. (**A**) 3D structure of *S. cerevisiae* frataxin homologue (YFH, PDB entry: 2GA5). (**B**) Proposed model of CreFH1. The structure of the CreFH1 was obtained by homology modeling using the @TOME v.3 Platform [60] and 2GA5 as template. The α-helices are shown in red and the β-sheets in light blue. (**C**) Electrostatic potential surface representation of CreFH1. Red and blue show negative and positive potentials, respectively. The protein has the same orientation as the ribbon structure showed in Figure 2B. (**D**) Superposed amino acid residues from the acidic patch of YFH (red) and CreFH1 (blue). Superposition of the 3D structures of YFH and CreFH1 was made using SuperPose server v. 1.0 [61].

To understand the structure of CreFH1 in more detail, a homology model was built as described in the “Methods” section, using the 3D structure of YFH (PDB entry: 2GA5) as a template. Analysis using ProSA-web server [52] showed a Z-score of −6.34, and the analysis using Verify 3D [53] showed that 95% of the residues had a score >0.2, indicating that the CreFH1 model is of good quality [54,55,56,57]. The monomeric CreFH1 structure exhibited a fold similar to YFH, with both α1- and α2-helix and β1 to β6-sheets conserved (Figure 2A,B). In addition, we found that the conserved N-terminal acidic residues in the α1/β1 regions of CreFH1 generate a negatively charged surface, as reported for other homologs (Figure 2C) [8,47]. These carboxylate side chains of aspartic and glutamic residues could serve as ligands for metal binding, as in many Fe-binding proteins, suggesting that these regions could be involved in this process (Figure 2D) [8]. Moreover, these amino acid residues may also participate in electrostatic interactions that could mediate protein–protein interactions. It was shown that electrostatic interactions play a more important role in protein binding than they do in folding [58,59].

### 2.2. Cloning, Expression and Purification of CreFH1

**Figure 3:**
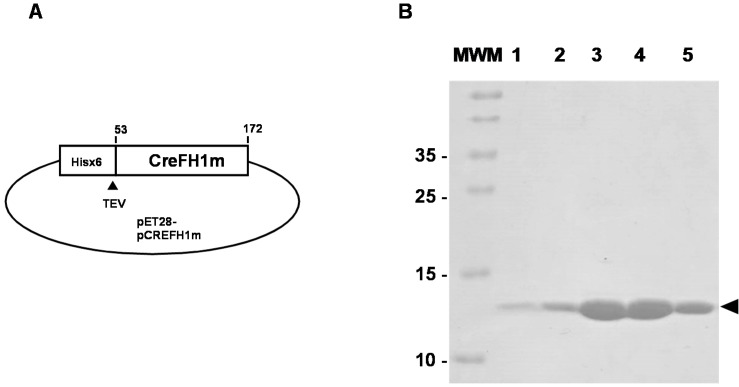
Cloning, expression and purification of CreFH1. (**A**) Schematic representation of plasmid pCREFH1m used to transform competent *E. coli* cells to express CreFH1 mature protein (from aa. 53 to 172) with a Hisx6 tag and a TEV protease cleavage site. (**B**) SDS-PAGE analysis of protein fractions eluted from CreFH1 after digestion with TEV protease. Lane 1: 0.5 ug CreFH1-His; Lane 2: 1 ug CreFH1-His; Lanes 3–5: CreFH1 fractions after digestion with TEV protease.

To characterize CreFH1 protein, we cloned the DNA fragment containing the Cre12.g538350t1.1 mature sequence (360 bp, 120 amino acids) without the mitochondrial transit peptide in a pET28 vector containing a His_6_ tag and a TEV cleavage site in the N-terminal region to generate the pCREFH1m plasmid (Figure 3A). The protein was expressed in *E. coli* (DE3) RIL cells and purified by a single purification step using Ni^2+^ affinity chromatography on a HiTrap chelating column. After elution, the protein was treated with TEV protease and, subsequently, the protease was removed using a Ni^2+^ resin. Using this procedure, we have obtained about 0.5 mg of CreFH1 protein from 10 g of *E. coli* cells. The eluted protein fractions were analyzed by SDS-PAGE. Figure 3B shows the presence of a highly purified single protein band of around 14 kDa. This agrees with the calculated molecular mass of CreFH1 (13.56 kDa). The protein fractions were pooled and concentrated to >1 mg/mL. The identity of the protein was confirmed by tryptic digestion and MALDI-TOF MS/MS mass spectrometry (not shown).

### 2.3. Oligomerization with Metals

**Figure 4:**
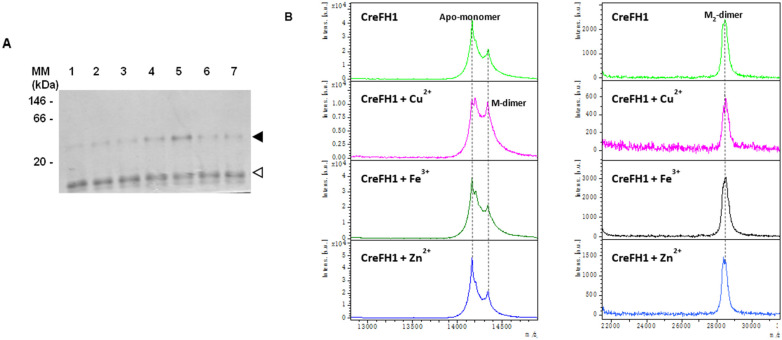
Analysis of CreFH1 oligomerization. (**A**) Analysis of CreFH1 oligomerization on 8% (*w*/*v*) native PAGE. Purified CreFH1 was incubated with metals (33 µM of Fe^3+^, Cu^2+^ or Zn^2+^) for 1 and 3 h, or with water as control. CreFH1 without metals (lane 1); CreF1 incubated with Fe^3+^ for 1 h (lane 2) or 3 h (lane 3); CreFH1 incubated with Cu^2+^ for 1 h (lane 4) or 3 h (lane 5); CreFH1 incubated with Zn^2+^ for 1 h (lane 6) or 3 h (lane 7). Each lane was loaded with 3 μg of protein. The white arrow indicates the CreFH1 monomer, and the black arrow corresponds to the dimer. MM is the native molecular mass marker (GE Healthcare Protein and Peptide Molecular Weight Markers). (**B**) ESI-MS spectra of CreFH1. Mass spectra were obtained after incubation of recombinant CreFH1 with Cu^2+^, Fe^3+^, Zn^2+^ or water (control, first panel) for 3 h. All the spectra showed the +1 charge state for the Apo-monomeric and M_2_-dimeric species. Notice that M-dimeric forms were observed at the +2 charge state (around 14,335 m/z), and that this species had a higher peak when CreFH1 was incubated with Cu^2+^. M denotes a metal.

One of the properties of human, yeast and bacteria frataxins is the ability to assemble and form oligomers spontaneously and/or in the presence of iron [28,62,63,64]. In order to evaluate the ability of CreFH1 to form oligomers, we evaluated the effect of the incubation of CreFH1 in the presence of different metal ions, such as Fe^3+^, Cu^2+^ and Zn^2+^. Thus, CreFH1 was incubated at 0 °C for 1 to 3 h in the presence of 33 µM of Fe^3+^, Cu^2+^ or Zn^2+^, and then analyzed by native PAGE (Figure 4A). We found that in the absence of metals, CreFH1 oligomerized in a low proportion, forming a dimeric structure of about 28 kDa. In the presence of metals, we observed a higher extent of CreFH1 oligomerization and also the formation of dimers. Interestingly, the highest dimer production (about 40% of the total protein) was observed in the presence of Cu^2+^. This is consistent with previous results obtained in our laboratory, where the frataxin homologs from *Arabidopsis thaliana* and *Zea mays* oligomerized when incubated with Cu^2+^ [35,65]. Oligomerization was also detected in the presence of Zn^2+^ and Fe^3+^; however, only about 22% of the protein was found as a dimer, while 78% remained as monomer (Figure 4A).

Recombinant CreFH1 proteins incubated with the different metals were also analyzed by ESI-MS, and the previous results were confirmed (Figure 4B). In all conditions tested, a major apo-monomeric species at m/z of 14,167 appeared, together with a smaller presence of a monometalated dimeric species with a MW of 14,335. The first peak corresponded to the methionine loss (MW 131) of the 14.3 kDa CreFH1 recombinant protein—a common modification in heterologous expression. The MW of the second peak can only be assigned to a dimer. It has the molecular weight of the whole protein plus half the mass of a metal (most probably Cu with an average mass of 63.55). This corresponds to the +2 charge state of a monometalated dimer, because a monomer cannot incorporate half a metal. Coincident with the native PAGE results, when CreFH1 was incubated with Cu^2+^, this dimeric peak had a stronger intensity. In the second panel, we observed additional dimeric forms with an MW of 28,445, which can be assigned to the methionine loss of two monomers plus two metals.

**Figure 5:**
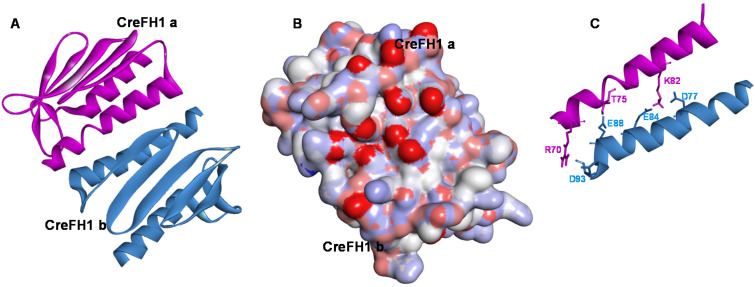
Haddock structure of dimeric CreFH1 with the highest score and lowest energy. (**A**) Ribbon structure representation of the CreFH1a:CreFH1b dimeric complex. (**B**) Electrostatic potential surface representation of CreFH1 dimer. Red and blue show negative and positive potentials, respectively. The complex has the same orientation as the ribbon structure shown in Figure 5A. (**C**) α1-helix segments from both subunits of the CreFH1 dimer showing the amino acid residues that would participate in the interaction.

The dimerization of CreFH1 (CreFH1a:CreFH1b complex) was analyzed in greater depth by docking studies using Haddock 2.4 [66]. Results show that the most efficient energy model is the one shown in Figure 5, with a score of −56.9 ± 15.6, suggesting that it is a good model. We found that the main amino acids possibly involved in the stabilization of the dimeric structure were mainly the acidic residues belonging from the α1 and β1 regions, such as Asp77, Glu84, Glu88 and Asp93, which could have interacted in electrostatic interactions with basic residues from the other chain, such as Arg70 and Lys82. In addition, there was a hydrogen bond interaction between Glu88 and Thr75, which also stabilized the dimer (Figure 5C). It is important to note that most of the acidic amino acids in the α1 and β1 regions are highly conserved among the frataxins of different organisms (see Figure 1B).

### 2.4. Attenuation of Fenton Reaction by CreFH1

Previous studies demonstrated that frataxin from *S.* *cerevisiae* and *Arabidopsis thaliana* has the ability to attenuate Fenton reaction, decreasing the oxidative damage induced by iron [15,36]. Based on this information, it was proposed that frataxin could act as an iron chaperone, attenuating metal-induced oxidative stress. In order to evaluate the existence of this activity in CreFH1, the inhibition of the production of malondialdehyde was measured spectrophotometrically at 532 nm.

**Figure 6:**
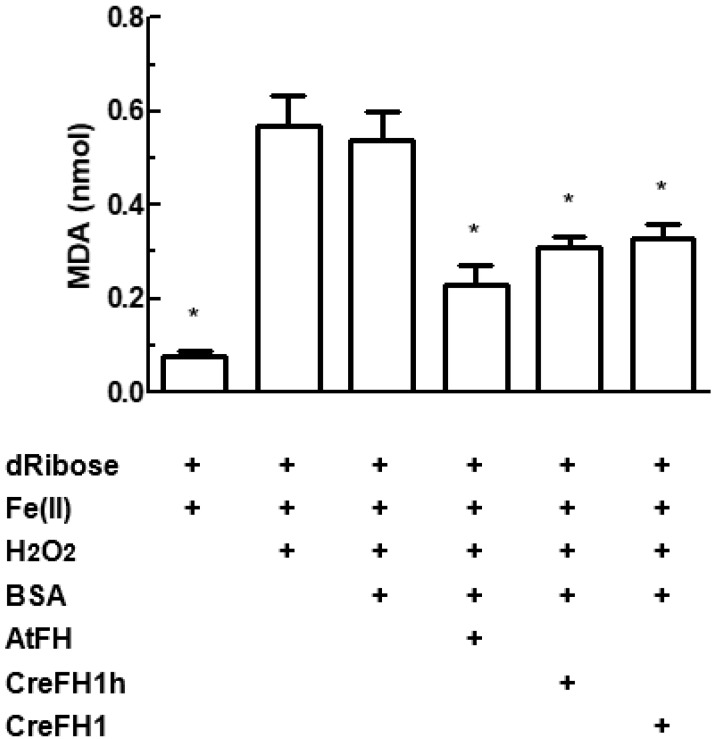
Measurement of the oxidative degradation of 2-deoxyribose. The reaction mixture was incubated in the presence or absence of CreFH1, CreFH-His or AtFH. The concentrations of the different compounds and measurement conditions are described in Materials and Methods. Asterisks indicate a statistically different results (* *p* < 0.05).

In the presence of dRibose, Fe^2+^ and H_2_O_2_, with or without BSA, about 0.55 nmol of malondialdehyde was obtained (Figure 6). However, in the presence of CreFH1, the production of malondialdehyde decreased about 40% compared with the control without the protein. Similar results were obtained in the presence of CreFH1h, containing the N-terminal His_6_ tag, indicating that the histidine residues did not affect the attenuation reaction. AtFH was used as a positive control, showing a decrease of around 50% in the amount of malondialdehyde produced (Figure 6), as reported previously [36]. Results showed that CreFH1 inhibited the production of malondialdehyde, attenuating Fenton’s reaction, suggesting that it could be involved in the protection against metal-induced oxidative stress.

### 2.5. Effect of Metals and H_2_O_2_ in E. coli Overexpressing AtFH and CreFH1

**Figure 7:**
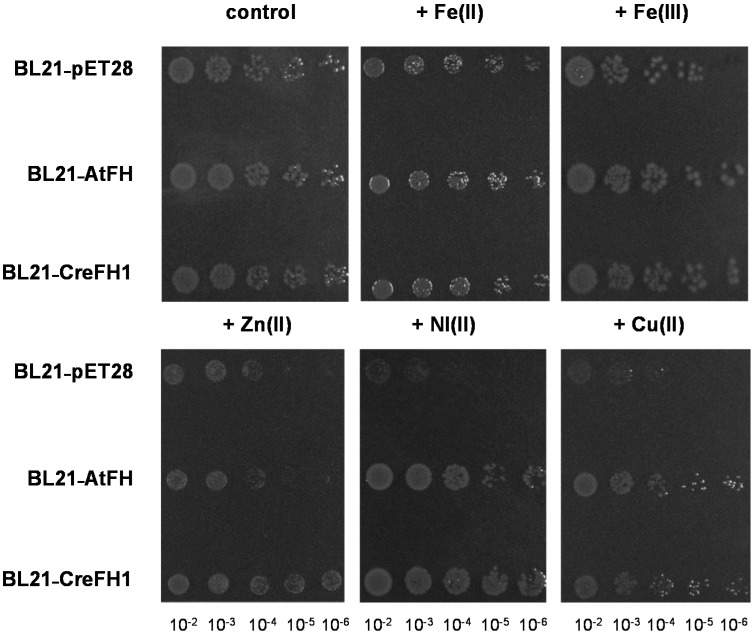
Effect of different metals on *E. coli* cells overexpressing CreFH1. *E. coli* cells transformed with the empty vector; AtFH and CreFH1 were spotted in 1/10 serial dilutions on LB agar plates supplemented with kanamycin (control), Fe(NH_4_)_2_(SO_4_)_2_ 2 mM, Fe(III) EDTA 1 mM, Zn_2_SO_4_ 1 mM, NiCl_2_ 1 mM and CuSO_4_ 1 mM. Cells were incubated overnight at 37 °C.

In order to study the influence that algae frataxin would have on metal metabolism, we tested the effect of frataxin overexpression on the restoration of the normal growth phenotype of *E. coli* cells. Thus, *E. coli* BL21-RIL cells overexpressing AtFH or CreFH1, or transformed with the empty pET28 vector as a negative control, were plated in LB agar supplemented with kanamycin and different metals. In the presence of Cu^2+^, Zn^2+^ and Ni^2+^, cells expressing AtFH or CreFH1 showed better growth than the negative control, while this effect was lower in the presence of Fe^2+^ or Fe^3+^ (Figure 7). In contrast, the overexpression of AtFH or CreFH1 had no effect on cell growth when Cr and As were supplemented (not shown).

**Figure 8:**
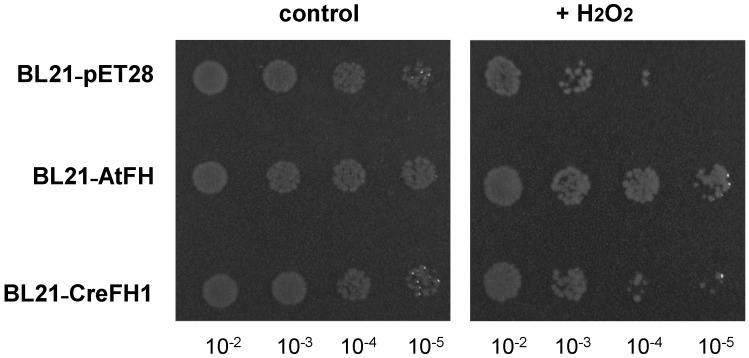
Effect of H_2_O_2_ on *E. coli* cells overexpressing CreFH1. BL21-RIL cells transformed with the empty vector; AtFH and CreFH1 were incubated for 1 h with 20 mM H_2_O_2_. As a negative control, all strains were incubated without H_2_O_2_. An amount of 5 μL of each culture (1/10 serial dilutions) was spotted on LB agar plates supplemented with kanamycin and incubated overnight at 37 °C.

Furthermore, in order to evaluate the role of CreFH1 in protection against oxidative stress, we also evaluated the restoration of normal growth of *E. coli* cells overexpressing frataxin homologues in the presence of hydrogen peroxide. Figure 8 shows that both groups of *E. coli* cells, those expressing AtFH and those expressing CreFH1, grew better than the control; however, the effect was more evident in the cells expressing AtFH. Our data agree with Fenton’s oxidative degradation results, suggesting a protective role of CreFH1 against H_2_O_2_-induced oxidative stress.

## 3. Discussion

Frataxin is a highly conserved protein during evolution, and is necessary for the proper functioning of mitochondria in eukaryotic organisms. Much work has been undertaken on the characterization of frataxin in humans because its deficiency is the cause of the autosomal recessive disease Friedreich’s ataxia [1,2,46]. Likewise, there are several reports regarding yeast that show that this protein has an essential role in iron metabolism, participating in the synthesis of Fe-S clusters and heme groups in mitochondria [2,14,15,48,67]. Although there are several works on the characterization of frataxins in different organisms, such as bacteria, plants, yeasts and humans, there is little information on these proteins in photosynthetic organisms, such as algae [2,9,32,33,35,47,68]

In our laboratory, we have reported the characterization of frataxin homologs from Arabidopsis and maize plants [32,33,35,36]. We proposed that plant frataxin is involved in the synthesis of Fe-S and heme groups in mitochondria [36]. In addition, we recently reported that AtFH shows ferrochelatase activity, which would confirm its participation in heme metabolism [17]. Furthermore, we recently showed that AtFH interacts with other proteins, such as AtNFS1 and AtIDS11, forming a multiprotein complex, as proposed in other organisms [29]. The AtNFS1-AtISD11-AtFS complex modulates the desulfurase activity of AtNFS1, and could also be important in mitigating oxidative damage in plant mitochondria, suggesting that it would have an important role in the early stages of Fe-S cluster synthesis in the organelle [29,69]. Other studies showed that frataxins from Arabidopsis and corn would have dual localization in mitochondria and chloroplasts [34,35]. Although maize was the first organism where the presence of two frataxins located in both organelles was described, there are other species where the presence of more than one frataxin gene was reported, such as *Glycine max* (soybean) and *Sorghum bicolor*. Thus, it was proposed that plant frataxins would have a relevant role not only in the correct function of plant mitochondria but also in that of chloroplasts [34,35].

In this study, we identified the presence of a gene that codes for a frataxin homolog in *C. reinhardtii* (CreFH1). We found that the *CreFH1* gene encodes a 172 aa protein that presents significant identity with respect to other eukaryotic frataxin homologues (between 41% and 51%). The main difference is in the N-terminus, which would code for a transit peptide most probably targeting mitochondria, and which would result in a mature protein of 120 amino acids.

The proposed 3D model of CreFH1 contains an N-terminal alpha helix, α1, five antiparallel β-sheet segments, two minor β-sheet segments and a C-terminal alpha helix α2. These secondary structures are similar to those reported for other frataxins, such as those from *E. coli* [50], *S. cerevisiae* [48] and humans [47]. In addition, we found that the conserved N-terminal acidic residues in the α1/β1 regions of CreFH1 generated a negatively charged surface, as reported for other homologs (Figure 2B) [8,47]. These carboxylate side chains of aspartic and glutamic residues serve as ligands for metal binding in many Fe-binding proteins, suggesting that these regions could be involved in this process [8]. Moreover, these amino acid residues could also participate in electrostatic interactions that mediate protein–protein interactions. It was shown that electrostatic interactions play a more important role in protein binding than they do in folding [58,59].

The small-scale synthesis of the protein and its subsequent purification showed the presence of a single protein band, confirmed by SDS-PAGE and MALDI-TOF peptide fingerprint analysis. However, while CreFH1 showed a 3D structure similar to other homologues, there were some structural and biochemical differences. The ESI-MS spectra and PAGE results showed that CreFH1 had the ability to form dimers, especially in the presence of cations, such as Fe^3+^ and Cu^2+^. The presence of dimers was not altered by the addition of DTT (data not shown), as occurs with the maize frataxins, ZmFHs [35,57]. ZmFHs change their oligomerization state in the presence of DTT (with a higher proportion of the monomeric form) due to the presence of a C-terminal cysteine residue, conserved in most plant frataxins (C193 in ZmHF1, C194 in ZmFH2 and C180 in AtFH). This residue is oxidized by metals forming dimers connected by disulfide bridges, which are reduced by DTT rendering the frataxin monomer. However, this residue is replaced by a valine (V165) in CreFH1, which is consistent with the lack of effect of DTT on the oligomerization state of this protein.

The presence of a peak in the mass spectrum corresponding to the monomer is in agreement with what was observed in the native PAGE analysis. As mentioned above, CreFH1 has the ability to partially oligomerize in the absence of metals; however, we found that the presence of Fe, or more especially of Cu, increased the proportion of the dimeric forms of the protein. Our data are in agreement with the properties of other frataxins, such as YFH1 from *S. cerevisiae* and CyaY from *E. coli*, which do not aggregate in vivo, but form multimers in vitro after the addition of Fe [70,71].

From the 3D structure of the *E. coli* CyaY, it was postulated that there would be three possible metal-binding sites [72]. Sites 2 and 3 are poorly conserved in CreFH;, however, it was postulated that there are four residues possibly involved in Fe binding at site 1, a His7 of one subunit, with the Glu19, Asp22 and Asp23 residues of another subunit [72]. The first three residues are conserved in CreFH1 (His69, Glu81, Glu84), while Asp23 is replaced by an alanine (Ala83) in CreFH1. The model of the CreFH1a:CreFH1b dimer obtained by molecular docking was in agreement with these data, and shows that these residues would be at the interaction interface between both subunits. On the other hand, the high number of acidic residues present at the interface between the CreFH1 monomers could have an essential role in stabilizing the dimeric structure, as described for the CyaY and HF oligomers [72]. In addition, another seven residues (Ser4, Glu5, Phe6, Arg20, Leu21, Trp24 and Asp31) of CyaY, whose NMR spectra are altered by titration with iron (with one or two atoms, with ferrous and ferric ions) are conserved or conservatively substituted in CreFH1 (Asn66, Asp67, Tyr68, Lys82, Leu83, Tyr86 and Asp96 respectively) [8]. The conservation of amino acids directly affected by the presence of Fe, or those in which a mutation inhibits Fe binding or affects the formation of Fe-S centers in pathway proteins, suggests that CreFH1 behaves similarly to CyaY, although it remains to collect more experimental evidence.

Hydrogen peroxide, as with many metals, is known to be highly oxidizing and toxic to all organisms, and these are protected by the combined action of peroxidases and catalases, which rapidly degrade it [73]. When the H_2_O_2_ scavenging capacity, or the ability to tolerate high metal concentrations, is exceeded, there are metabolic defects that can lead to enzyme damage, especially iron and sulfur enzymes, in addition to DNA damage [74].

When we supplemented *E. coli* cells with CreFH1, we observed that the cells had a higher growth capacity in the presence of H_2_O_2_ and/or metals, such as Cu, Fe, Zn and Ni. Furthermore, in vitro experiments showed that CreFH1 could attenuate the Fenton oxidative reaction. These results are in agreement with the evidence of a protective role of CreFH1 against oxidizing agents in algae, as has been described for other frataxin homologues [6,32,35,36]. From our results, we can postulate that CreFH1 would be involved in the homeostasis of different metals, and therefore, would play an important role in acclimatization to oxidative stress in algae.

Thus, this is the first study on the structural and functional characterization of an algae frataxin. CreFH1 has an overall structure similar to described frataxins from other organisms. This is reflected in the analysis of its amino acid sequence and its 3D structure. This protein has some particular characteristics, such as the possibility of increasing the degree of dimerization in the presence of metals. It was also shown to be effective in attenuating oxidative stress in *E. coli* cells. From our data, we can confirm that CreFH1 is a functional protein in *C. reinhardtii* and that it possibly fulfills similar functions in this organism to those described for other frataxins, as a Fe donor, modulating the activity of Fe-S proteins and in the protection against oxidative stress.

## 4. Materials and Methods

### 4.1. Algal Strains and Culture Conditions

*Chlamydomonas reinhardtii* wild-type strain CC-1690 was obtained from the Instituto de Biología de la Pontificia Universidad Católica de Valparaíso. Cells were grown at 24 °C in a modified TAP medium: 2.42 g Tris-base; 25 mL of TAP salts solution (7 mM NH_4_Cl, 0.83 mM MgSO_4_.7H_2_O, 0.45 mM CaCl_2_.2H_2_O); 1 mL of Pi solution (1.65 mM K_2_HPO_4_, 1.05 mM KH_2_PO_4_); 1 mL of Hunter solution (0.13 mM Na_2_EDTA.2H_2_O, 0.13 mM ZnSO_4_.7H_2_O, 0.18 mM H_3_BO_3_, 0.04 mM MnCl_2_.4H_2_O, 0.033 mM FeSO_4_.7H_2_O, 0.12 mM CoCl_2_.6H_2_O, 0.01 mM CuSO_4_.5H_2_O, 4.5 uM (NH_4_)6MoO_3_); 1 mL HAc and H_2_O (up to 1 L). The pH was adjusted to pH = 7. Cells were incubated with moderate shaking (120 rpm) and illumination with a 16 h light/8 h dark cycle (100 µmol s^−1^m^2^ photosynthetic active radiation) in Erlenmeyer flasks.

### 4.2. Cloning, Expression and Purification of the Chlamydomonas Reinhardtii Frataxin Homolog CreFH1

The genomic, transcript and CDS sequences of the *CreFH1* gene (Cre12.g538350t1.1) from *C. reinhardtii* were obtained from Phytozome v12 [42]. Total RNA from a *C. reinhardtii* culture grown in TAP medium for 8 days was extracted with Trizol reagent (Invitrogen, Carlsbad, CA, USA), yielding about 100 mg of tissue/mL. cDNA was obtained by reverse transcription from 2 µg of RNA. The reaction mix contained 200 U of M-MLV RT (Promega, Fitchburg, WI, USA), 15 µg of Random Primers pd(N) (Amersham Biosciences, UK), 0.5 mM dNTPs, 1X buffer 5X M-MLV (Promega, Fitchburg, WI, USA), 25 U RNAse Inhibitor (Promega, Fitchburg, WI, USA) and H_2_O DEPC to complete 25 µL. The reaction was incubated at 42 °C for 1 h.

*CreFH1* mature CDS without the transit peptide coding sequence of 52 amino acids was cloned into a pET28 plasmid (modified with a TEV cleaving site before the MCS) [75] to produce the plasmid pCREFH1. Primers used to amplify the sequence by PCR were creffw (5′ AATGAATTCAATGGCACGGAGAG 3′) and crefrv (5′ ATTCTCGAGTCACTCCAGCTGC 3′, EcoRI and XhoI restrictions sites are underlined).

*E. coli* BL21-RIL cells harboring pCREFH1m plasmid were grown at 37 °C in LB medium containing Kanamycin (30 mg/mL) to an OD_600_ = 0.5. Protein production was induced by the addition of 1 mM IPTG and subsequent incubation for 3 h at 30 °C. Cells were harvested by centrifugation, resuspended in 20 mM Tris-HCl (pH 7.5), disrupted by sonication and centrifuged at 10,000× *g* for 15 min at 4 °C.

For the purification of CreFH1, the soluble extract was loaded into a HiTrap chelating column (GE Healthcare Bio-Sciences Corp, Uppsala, Sweden) and the column was washed with 10 mL of 20 mM Tris-HCl (pH 7.5) and 20 mM imidazole. The recombinant protein was eluted with an imidazole gradient (20 mM to 400 mM) in 20 mM Tris-HCl (pH 7.5). Purified fractions were diluted with 20 mM Tris-HCl (pH 7.5) and 20% (*v*/*v*) glycerol and stored at −40 °C. In some experiments, the purified fractions containing CreFH1 were treated with TEV protease in a reaction buffer containing 50 mM Tris–HCl, pH 8.0, 0.5 mM EDTA and 1 mM DTT, at 4 °C overnight. TEV protease was removed by incubation with a Ni^2+^ resin, and the CreFH1 was recovered in the soluble fraction [76].

### 4.3. Native and SDS-PAGE Analysis

Native PAGE was carried out in Mini Protean II Cell (BioRad, Hercules, CA, USA) using 8%, 10% and 12% (*w*/*v*) acrylamide/bisacrylamide separating gels with 8% (*w*/*v*) stacking gels as described [77]. Purified recombinantCreFH1 samples were incubated at room temperature for 1 h or 3 h with metals (Fe(III)-EDTA, ZnSO_4_, CuSO_4_). As a negative control, CreFH1 was incubated with distilled water. After incubation, samples were mixed with loading buffer [62.5 mM Tris-HCl (pH 6.8), 25% (*v*/*v*) glycerol, 1% (*w*/*v*) bromophenol blue]. Three μg of protein samples were loaded per well. The electrophoresis was performed with a buffer containing 25 mM Tris-HCl (pH 8.3) and 192 mM glycine. After placing the electrophoresis cell on ice, a voltage of 100 V was applied for 1 h, followed by a voltage of 150 V until the electrophoretic run was completed. Gels were stained with a solution containing 0.3% (*w*/*v*) Coomassie Brilliant Blue (R-250), 45% (*v*/*v*) methanol and 10% (*v*/*v*) acetic acid.

Purified recombinant CreFH1 was analyzed by SDS-PAGE using 12% (*w*/*v*) gels, as described by Laemmli [78] in a Mini Protean II Cell (Bio-Rad). After electrophoresis, gels were stained by Coomassie Brilliant Blue. Total protein concentrations were determined by Bradford method, measuring absorbance at 595 nm and BSA as a reference standard [79]. The relative protein levels were determined by densitometric analysis using GelPro analyzer program (Media Cybernetics, Bethesda, MD, USA).

### 4.4. ESI-TOF MS Molecular Mass Determinations

All samples were analyzed under the following conditions: 20 μL of protein solution injected at 40 μL min^−1^; a capillary counterelectrode voltage of 5 kV; desolvation temperature between 90 and 110 °C. The carrier buffer was a 5:95 mixture of acetonitrile:ammonium acetate/ammonia (15 mM, pH 7.0). The equipment used was a Micro TOF-Q instrument (Bruker, Billerica, MA, USA) interfaced with an Agilent Series 1200 HPLC pump, both controlled using the Compass Software. Calibration was attained with ESI-L Low Concentration Tuning Mix (Agilent Technologies, Santa Clara, CA, USA). All the spectra were recorded at least 3 times in order to ensure the reproducibility of the experiments. In all cases, the spectra were perfectly matched among different experiments.

### 4.5. Oxidative Degradation Assays

The ability of CreFH1 to attenuate Fenton’s reaction was determined by measuring the inhibition of the production of malondialdehyde after the addition of thiobarbituric acid, as previously described [36]. Briefly, a mixture of 15 µM Fe(II), 8 µM H_2_O_2_ and 5 mM 2-deoxyribose (Fluka Analytical) was incubated in Hepes-KOH (pH 7.0) in the absence or in the presence of recombinant CreFH1. As a positive control, recombinant AtFH was used [36]. The final volume of the reaction was 100 µL. After 30 min of incubation at 25 °C, 100 µL of 1% (*w*/*v*) thiobarbituric acid and 100 µL of 4% (*v*/*v*) phosphoric acid were added to the reaction mixture. The samples were incubated for 10 min at 100 °C. After cooling on ice, 75 µL of 10% (*w*/*v*) SDS was added to each tube. The amount of malondialdehyde was determined spectrophotometrically, by measuring absorbance at 532 nm.

### 4.6. Effect of Metals and H_2_O_2_ on E. coli Cells Overexpressing AtFH or CreFH1

*E. coli* BL21-RIL strains were transformed with pET28-AtFH, pCREFH1m and the pET28 empty vector. Cells were grown in LB medium containing Kanamycin (30 mg/mL) at 37 °C to an OD_600_ = 0.5. Protein production was induced by the addition of 0.5 mM IPTG and subsequent incubation for 3 h at 30 °C. After induction, OD_600_ was measured, and the cultures were diluted to OD_600_ = 1.

To analyze the effect of metals, 1/10 serial dilutions of the *E. coli* cultures were plated in LB agar medium supplemented with Kanamycin (30 mg/mL) and the following metals: Fe(NH_4_)_2_(SO_4_)_2_ 2 mM, Fe(III)-EDTA 2 mM, CuSO_4_ 1 mM, Zn_2_SO_4_ 1 mM, NiCl_2_ 1 mM y (NH_4_)_2_CrO_4_ 100 µM. To analyze the effect of H_2_O_2_, 1 mL of each culture was incubated with 20 mM H_2_O_2_ for 1 h. As a negative control, all cultures were incubated without H_2_O_2_. 1/10 serial dilutions were plated in LB agar medium supplemented with Kanamycin (30 mg/mL). The cells were grown overnight at 37 °C, and then photographed.

### 4.7. Additional Methods

The 3D structural models were obtained at the @TOME v.3Platform [60,80]. Models were evaluated with Verify3D structure analysis program [53,54,55]. Superpositions of protein structures were analyzed using the SuperPose server v. 1.0 [61,81]. Docking predictions were made using HADDOCK 2.4 [66] with default settings and using as active residues the conserved ones that would be involved in the oligomerization of the human, yeast and bacterial frataxins [66].

## Data Availability

Not applicable.

## References

[1] Campuzano V., Montermni L., Molto M.D., Pianese L., Cossee M., Cavalcanti F., Monros E., Rodius F., Duclos F., Monticelli A. (1996). Friedreich’s ataxia: Autosomal recessive disease caused by an intronic GAA triplet repeat expansion. Science.

[2] Babcock M., de Silva D., Oaks R., Davis-Kaplan S., Jiralerspong S., Montermini L., Pandolfo M., Kaplan J. (1997). Regulation of mitochondrial iron accumulation by Yfh1p, a putative homolog of frataxin. Science.

[3] Isaya G., O’Neill H.A., Gakh O., Park S., Mantcheva R., Mooney S.M. (2004). Functional studies of frataxin. Acta Paediatr..

[4] Pastore A., Puccio H. (2013). Frataxin: A protein in search for a function. J. Neurochem..

[5] Martin M., Colman M.J., Gomez-Casati D.F., Lamattina L., Zabaleta E.J. (2009). Nitric oxide accumulation is required to protect against iron-mediated oxidative stress in frataxin-deficient Arabidopsis plants. FEBS Lett..

[6] Ristow M., Pfister M.F., Yee A.J., Schubert M., Michael L., Zhang C.Y., Ueki K., Michael M.D., Lowell B.B., Kahn C.R. (2000). Frataxin activates mitochondrial energy conversion and oxidative phosphorylation. Proc. Natl. Acad. Sci. USA.

[7] Bulteau A.L., O’Neill H.A., Kennedy M.C., Ikeda-Saito M., Isaya G., Szweda L.I. (2004). Frataxin acts as an iron chaperone protein to modulate mitochondrial aconitase activity. Science.

[8] Bencze K.Z., Kondapalli K.C., Cook J.D., McMahon S., Millan-Pacheco C., Pastor N., Stemmler T.L. (2006). The structure and function of frataxin. Crit. Rev. Biochem. Mol. Biol..

[9] Busi M.V., Gomez-Casati D.F. (2012). Exploring frataxin function. IUBMB Life.

[10] Gomez-Casati D.F., Busi M.V., Pagani M.A. (2018). Plant Frataxin in Metal Metabolism. Front. Plant Sci..

[11] Schoenfeld R.A., Napoli E., Wong A., Zhan S., Reutenauer L., Morin D., Buckpitt A.R., Taroni F., Lonnerdal B., Ristow M. (2005). Frataxin deficiency alters heme pathway transcripts and decreases mitochondrial heme metabolites in mammalian cells. Hum. Mol. Genet..

[12] Napoli E., Morin D., Bernhardt R., Buckpitt A., Cortopassi G. (2007). Hemin rescues adrenodoxin, heme a and cytochrome oxidase activity in frataxin-deficient oligodendroglioma cells. Biochim. Biophys. Acta.

[13] Maliandi M.V., Busi M.V., Turowski V.R., Leaden L., Araya A., Gomez-Casati D.F. (2011). The mitochondrial protein frataxin is essential for heme biosynthesis in plants. FEBS J..

[14] Muhlenhoff U., Richhardt N., Gerber J., Lill R. (2002). Characterization of iron-sulfur protein assembly in isolated mitochondria: A requirement for ATP, NADH, and reduced iron. J. Biol. Chem..

[15] Park S., Gakh O., Mooney S.M., Isaya G. (2002). The ferroxidase activity of yeast frataxin. J. Biol. Chem..

[16] Yoon T., Cowan J.A. (2003). Iron-sulfur cluster biosynthesis. Characterization of frataxin as an iron donor for assembly of [2Fe-2S] clusters in ISU-type proteins. J. Am. Chem. Soc..

[17] Armas A.M., Balparda M., Terenzi A., Busi M.V., Pagani M.A., Gomez-Casati D.F. (2019). Ferrochelatase activity of plant frataxin. Biochimie.

[18] Du J., Zhou Y., Li Y., Xia J., Chen Y., Chen S., Wang X., Sun W., Wang T., Ren X. (2020). Identification of Frataxin as a regulator of ferroptosis. Redox Biol..

[19] Chen O.S., Hemenway S., Kaplan J. (2002). Inhibition of Fe-S cluster biosynthesis decreases mitochondrial iron export: Evidence that Yfh1p affects Fe-S cluster synthesis. Proc. Natl. Acad. Sci. USA.

[20] Gerber J., Muhlenhoff U., Lill R. (2003). An interaction between frataxin and Isu1/Nfs1 that is crucial for Fe/S cluster synthesis on Isu1. EMBO Rep..

[21] Tsai C.L., Barondeau D.P. (2010). Human frataxin is an allosteric switch that activates the Fe-S cluster biosynthetic complex. Biochemistry.

[22] Turowski V.R., Busi M.V., Gomez-Casati D.F. (2012). Structural and functional studies of the mitochondrial cysteine desulfurase from Arabidopsis thaliana. Mol. Plant.

[23] Fox N.G., Yu X., Feng X., Bailey H.J., Martelli A., Nabhan J.F., Strain-Damerell C., Bulawa C., Yue W.W., Han S. (2019). Structure of the human frataxin-bound iron-sulfur cluster assembly complex provides insight into its activation mechanism. Nat. Commun..

[24] Cai K., Frederick R.O., Tonelli M., Markley J.L. (2018). Interactions of iron-bound frataxin with ISCU and ferredoxin on the cysteine desulfurase complex leading to Fe-S cluster assembly. J. Inorg. Biochem..

[25] Saha P.P., Srivastava S., Kumar S.K.P., Sinha D., D’Silva P. (2015). Mapping Key Residues of ISD11 Critical for NFS1-ISD11 Subcomplex Stability: Implications in the development of mitochondrial disorder, COXPD19. J. Biol. Chem..

[26] Shan Y., Napoli E., Cortopassi G. (2007). Mitochondrial frataxin interacts with ISD11 of the NFS1/ISCU complex and multiple mitochondrial chaperones. Hum. Mol. Genet..

[27] Yoon T., Cowan J.A. (2004). Frataxin-mediated iron delivery to ferrochelatase in the final step of heme biosynthesis. J. Biol. Chem..

[28] Park S., Gakh O., O’Neill H.A., Mangravita A., Nichol H., Ferreira G.C., Isaya G. (2003). Yeast frataxin sequentially chaperones and stores iron by coupling protein assembly with iron oxidation. J. Biol. Chem..

[29] Armas A.M., Balparda M., Terenzi A., Busi M.V., Pagani M.A., Gomez-Casati D.F. (2020). Iron-Sulfur Cluster Complex Assembly in the Mitochondria of Arabidopsis thaliana. Plants.

[30] Gakh O., Park S., Liu G., Macomber L., Imlay J.A., Ferreira G.C., Isaya G. (2006). Mitochondrial iron detoxification is a primary function of frataxin that limits oxidative damage and preserves cell longevity. Hum. Mol. Genet..

[31] Karlberg T., Schagerlof U., Gakh O., Park S., Ryde U., Lindahl M., Leath K., Garman E., Isaya G., Al-Karadaghi S. (2006). The structures of frataxin oligomers reveal the mechanism for the delivery and detoxification of iron. Structure.

[32] Busi M.V., Zabaleta E.J., Araya A., Gomez-Casati D.F. (2004). Functional and molecular characterization of the frataxin homolog from Arabidopsis thaliana. FEBS Lett..

[33] Busi M.V., Maliandi M.V., Valdez H., Clemente M., Zabaleta E.J., Araya A., Gomez-Casati D.F. (2006). Deficiency of Arabidopsis thaliana frataxin alters activity of mitochondrial Fe-S proteins and induces oxidative stress. Plant J. Cell Mol. Biol..

[34] Turowski V.R., Aknin C., Maliandi M.V., Buchensky C., Leaden L., Peralta D.A., Busi M.V., Araya A., Gomez-Casati D.F. (2015). Frataxin Is Localized to Both the Chloroplast and Mitochondrion and Is Involved in Chloroplast Fe-S Protein Function in Arabidopsis. PLoS ONE.

[35] Buchensky C., Sanchez M., Carrillo M., Palacios O., Capdevila M., Dominguez-Vera J.M., Busi M.V., Atrian S., Pagani M.A., Gomez-Casati D.F. (2017). Identification of two frataxin isoforms in Zea mays: Structural and functional studies. Biochimie.

[36] Maliandi M.V., Busi M.V., Clemente M., Zabaleta E.J., Araya A., Gomez-Casati D.F. (2007). Expression and one-step purification of recombinant Arabidopsis thaliana frataxin homolog (AtFH). Protein Expr. Purif..

[37] Vazzola V., Losa A., Soave C., Murgia I. (2007). Knockout of frataxin gene causes embryo lethality in Arabidopsis. FEBS Lett..

[38] Hippler M., Redding K., Rochaix J.D. (1998). Chlamydomonas genetics, a tool for the study of bioenergetic pathways. Biochim. Biophys. Acta.

[39] Glaesener A.G., Merchant S.S., Blaby-Haas C.E. (2013). Iron economy in *Chlamydomonas reinhardtii*. Front. Plant Sci..

[40] Godman J., Balk J. (2008). Genome analysis of *Chlamydomonas reinhardtii* reveals the existence of multiple, compartmentalized iron-sulfur protein assembly machineries of different evolutionary origins. Genetics.

[41] Gomez-Casati D.F., Busi M.V., Barchiesi J., Pagani M.A., Marchetti-Acosta N.S., Terenzi A. (2021). Fe-S Protein Synthesis in Green Algae Mitochondria. Plants.

[42] Phytozome v12. https://phytozome.jgi.doe.gov/pz/portal.html.

[43] CD Search. https://www.ncbi.nlm.nih.gov/Structure/cdd/wrpsb.cgi.

[44] Marchler-Bauer A., Lu S., Anderson J.B., Chitsaz F., Derbyshire M.K., DeWeese-Scott C., Fong J.H., Geer L.Y., Geer R.C., Gonzales N.R. (2011). CDD: A Conserved Domain Database for the functional annotation of proteins. Nucleic Acids Res..

[45] TargetP-2.0 Server. http://www.cbs.dtu.dk/services/TargetP/.

[46] Cho S.J., Lee M.G., Yang J.K., Lee J.Y., Song H.K., Suh S.W. (2000). Crystal structure of Escherichia coli CyaY protein reveals a previously unidentified fold for the evolutionarily conserved frataxin family. Proc. Natl. Acad. Sci. USA.

[47] Dhe-Paganon S., Shigeta R., Chi Y.I., Ristow M., Shoelson S.E. (2000). Crystal structure of human frataxin. J. Biol. Chem..

[48] He Y., Alam S.L., Proteasa S.V., Zhang Y., Lesuisse E., Dancis A., Stemmler T.L. (2004). Yeast frataxin solution structure, iron binding, and ferrochelatase interaction. Biochemistry.

[49] Musco G., Stier G., Kolmerer B., Adinolfi S., Martin S., Frenkiel T., Gibson T., Pastore A. (2000). Towards a structural understanding of Friedreich’s ataxia: The solution structure of frataxin. Structure.

[50] Nair M., Adinolfi S., Pastore C., Kelly G., Temussi P., Pastore A. (2004). Solution structure of the bacterial frataxin ortholog, CyaY: Mapping the iron binding sites. Structure.

[51] Jpred 4. http://www.compbio.dundee.ac.uk/jpred/.

[52] ProSA-web server. https://prosa.services.came.sbg.ac.at/prosa.php.

[53] Verify 3D. https://servicesn.mbi.ucla.edu/Verify3D/.

[54] Bowie J.U., Luthy R., Eisenberg D. (1991). A method to identify protein sequences that fold into a known three-dimensional structure. Science.

[55] Luthy R., Bowie J.U., Eisenberg D. (1992). Assessment of protein models with three-dimensional profiles. Nature.

[56] Wiederstein M., Sippl M.J. (2007). ProSA-web: Interactive web service for the recognition of errors in three-dimensional structures of proteins. Nucleic Acids Res..

[57] Sippl M.J. (1993). Recognition of errors in three-dimensional structures of proteins. Proteins.

[58] Kundrotas P.J., Alexov E. (2006). Predicting 3D structures of transient protein-protein complexes by homology. Biochim. Biophys. Acta.

[59] Hu Z., Ma B., Wolfson H., Nussinov R. (2000). Conservation of polar residues as hot spots at protein interfaces. Proteins.

[60] @Tome v.3. http://atome.cbs.cnrs.fr/ATOME_V3/index.html.

[61] SuperPose server v. 1.0. http://superpose.wishartlab.com/.

[62] Cavadini P., O’Neill H.A., Benada O., Isaya G. (2002). Assembly and iron-binding properties of human frataxin, the protein deficient in Friedreich ataxia. Hum. Mol. Genet..

[63] Bou-Abdallah F., Adinolfi S., Pastore A., Laue T.M., Dennis Chasteen N. (2004). Iron binding and oxidation kinetics in frataxin CyaY of Escherichia coli. J. Mol. Biol..

[64] Gakh O., Adamec J., Gacy A.M., Twesten R.D., Owen W.G., Isaya G. (2002). Physical evidence that yeast frataxin is an iron storage protein. Biochemistry.

[65] Sanchez M., Palacios O., Buchensky C., Sabio L., Gomez-Casati D.F., Pagani M.A., Capdevila M., Atrian S., Dominguez-Vera J.M. (2018). Copper redox chemistry of plant frataxins. J. Inorg. Biochem..

[66] van Zundert G.C.P., Rodrigues J., Trellet M., Schmitz C., Kastritis P.L., Karaca E., Melquiond A.S.J., van Dijk M., de Vries S.J., Bonvin A. (2016). The HADDOCK2.2 Web Server: User-Friendly Integrative Modeling of Biomolecular Complexes. J. Mol. Biol..

[67] Seguin A., Bayot A., Dancis A., Rogowska-Wrzesinska A., Auchere F., Camadro J.M., Bulteau A.L., Lesuisse E. (2009). Overexpression of the yeast frataxin homolog (Yfh1): Contrasting effects on iron-sulfur cluster assembly, heme synthesis and resistance to oxidative stress. Mitochondrion.

[68] Noguera M.E., Roman E.A., Rigal J.B., Cousido-Siah A., Mitschler A., Podjarny A., Santos J. (2015). Structural characterization of metal binding to a cold-adapted frataxin. J. Biol. Inorg. Chem. JBIC A Publ. Soc. Biol. Inorg. Chem..

[69] Armas A.M., Balparda M., Turowski V.R., Busi M.V., Pagani M.A., Gomez-Casati D.F. (2019). Altered levels of mitochondrial NFS1 affect cellular Fe and S contents in plants. Plant Cell Rep..

[70] Adinolfi S., Trifuoggi M., Politou A.S., Martin S., Pastore A. (2002). A structural approach to understanding the iron-binding properties of phylogenetically different frataxins. Hum. Mol. Genet..

[71] Adamec J., Rusnak F., Owen W.G., Naylor S., Benson L.M., Gacy A.M., Isaya G. (2000). Iron-dependent self-assembly of recombinant yeast frataxin: Implications for Friedreich ataxia. Am. J. Hum. Genet..

[72] Ahlgren E.C., Fekry M., Wiemann M., Soderberg C.A., Bernfur K., Gakh O., Rasmussen M., Hojrup P., Emanuelsson C., Isaya G. (2017). Iron-induced oligomerization of human FXN81-210 and bacterial CyaY frataxin and the effect of iron chelators. PLoS ONE.

[73] Ravindra Kumar S., Imlay J.A. (2013). How Escherichia coli tolerates profuse hydrogen peroxide formation by a catabolic pathway. J. Bacteriol..

[74] Jang S., Imlay J.A. (2007). Micromolar intracellular hydrogen peroxide disrupts metabolism by damaging iron-sulfur enzymes. J. Biol. Chem..

[75] Peralta D.A., Araya A., Busi M.V., Gomez-Casati D.F. (2016). The E3 ubiquitin-ligase SEVEN IN ABSENTIA like 7 mono-ubiquitinates glyceraldehyde-3-phosphate dehydrogenase 1 isoform in vitro and is required for its nuclear localization in Arabidopsis thaliana. Int. J. Biochem. Cell Biol..

[76] Tropea J.E., Cherry S., Waugh D.S. (2009). Expression and purification of soluble His(6)-tagged TEV protease. Methods Mol. Biol..

[77] Gomez Casati D.F., Sesma J.I., Iglesias A.A. (2000). Structural and kinetic characterization of NADP-dependent, non-phosphorylating glyceraldehyde-3-phosphate dehydrogenase from celery leaves. Plant Sci..

[78] Laemmli U.K. (1970). Cleavage of structural proteins during the assembly of the head of bacteriophage T4. Nature.

[79] Bradford M.M. (1976). A rapid and sensitive method for the quantitation of microgram quantities of protein utilizing the principle of protein-dye binding. Anal. Biochem..

[80] Pons J.L., Labesse G. (2009). @TOME-2: A new pipeline for comparative modeling of protein-ligand complexes. Nucleic Acids Res..

[81] Maiti R., Van Domselaar G.H., Zhang H., Wishart D.S. (2004). SuperPose: A simple server for sophisticated structural superposition. Nucleic Acids Res..

